# Insulin-like Growth Factor and its Therapeutic Potential for Diabetes Complications - Mechanisms and Metabolic Links: A Review

**DOI:** 10.1900/RDS.2020.16.24

**Published:** 2021-05-01

**Authors:** Belete Biadgo, Workineh Tamir, Sintayehu Ambachew

**Affiliations:** 1 Department of Clinical Chemistry, School of Biomedical and Laboratory Sciences, College of Medicine and Health Sciences, University of Gondar, Ethiopia.; 2 Department of Medical Laboratory Science, College of Medicine and Health Sciences, Debre Markos University, Debre Markos, Ethiopia.

**Keywords:** insulin-like growth factor, diabetes complications, type 2 diabetes, insulin

## Abstract

**BACKGROUND:**

The insulin-like growth factor (IGF) system is an important system in normal physiological functioning of the body. In diabetes mellitus, alterations of IGF-binding protein (IGFBP) levels have been described, mainly in vascular complications.

**AIM:**

The aim of this review was to explore the role of the IGF system in reducing diabetes complications and its role as potential therapeutic target.

**RESULTS:**

IGF-1 plays a role in neuronal growth and developmental processes. Low concentrations of IGF-1 have been associated with neuropathy and other diabetes complications. Moreover, impaired IGF synthesis and function may result in cellular senescence and impaired vascular endothelial proliferation, adhesion, and integration. Of note, high IGF-1 bioavailability may prevent or delay the inception of diabetes-associated complications in diabetes patients. The mechanism of normal functioning IGF-1 is induced by increasing nitric oxide synthesis and potassium ion channel opening in cardiovascular physiology, which improves impaired small blood vessel function and reduces the occurrence of diabetes complications associated with reduced concentrations of IGF-1.

**CONCLUSIONS:**

IGF may be considered an alternative therapy for diabetes and diabetes-associated complications. Therefore, future studies should focus on the mechanism of action and therapeutic potential of IGFs in reducing the risk of development and progression of the disease in different clinical settings.

## 1 Introduction

The insulin-like growth factor (IGF) system is involved in the regulation of mammalian cell growth and differentiation, proliferation, and survival [1]. The system affects any of the other systems in our body. IGF-1 is a small protein consisting of 70 amino acids, with a molecular weight of 7.65 kilo Dalton, and the gene is located at chromosome 12q23 [1]. It is mainly produced by liver cells, but also by many other cells in our body [2].

The IGF system consists of:

- 2 cell-surface receptors (IGF-1R and IGF-2R)
- 2 ligands (IGF-1 and IGF-2)
- 6 high-affinity IGF-binding proteins (IGFBP-1 toIGFBP-6) [3, 4]
- Several associated IGFBP degrading protease enzymes.

**Figure 1.**
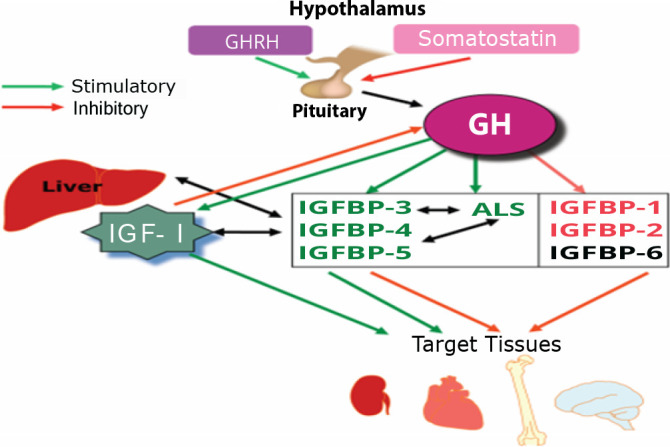
The multifaceted influence of GH through the IGF system. ALS: Acid labile subunit; GH: growth hormone; GHRH: GH releasing hormone; IGF: insulin-like growth factor; IGFBP: IGF-binding protein (adapted from Blum, *et al.*, [9])

The entire system is strongly controlled by a feedback loop involving growth hormones (GH) secreted by the pituitary, and GH production and secretion controlled by growth hormone-releasing hormone (GHRH) in the hypothalamus [4, 5] (Figure 1).

IGF-1 and IGFBP-3 are GH-dependent [6], while IGFBP-1 is insulin-regulated. IGFBP-1 production from the liver is significantly elevated during insulinopenia, and serum levels of bioactive IGF-1 are increased by insulin [7]. The production of IGFBP-3, -4, and -5 is stimulated by GH. IGFBP-3 is formed by the liver sinusoidal cells at the junction of the intravascular space. In circulation, IGF-1 is mainly bound to IGFBP-3, and this binary complex then binds to a large protein called the acid-labile subunit (ALS) to form a ternary complex [8, 9].

Insulin and IGF-1 are two related peptides with similar structure. They exercise their effects by interacting with their corresponding receptors, namely the insulin receptor and IGF-1R. The receptor-ligand interactions induce intracellular signaling cascades resulting in metabolic or mitogenic effects [10, 11] and are involved in regulation of metabolism. In contrast, IGF-1 overproduction in some pancreatic and non-pancreatic cancers has been linked to severe hypoglycemia [11].

Insulin encourages the constitutive secretion of IGF-1 from the liver. In turn, IGF-1 overruns insulin secretion even in normoglycemic situations [12]. Furthermore, a previous study showed that IGF-1 causes insulin activity and peripheral glucose utilizations to increase, hepatic glucose production to decrease, and lipid profiles in diabetes patients to improve [13].

IGFBP-1 is regulated mainly by insulin. It interacts with IGF-1 and IGF-2, and is used as a shuttle for IGFs to target tissues and regulate the action of free IGF-1. IGFPB-1 is regarded as the primary regulator of IGF-1 bioactivity and has an important part in the progression of diabetes and diabetes-related complications [14]. Lewitt *et al*. reported that the IGF system has an imperative pathophysiological role across a range of metabolic abnormalities, including obesity, insulin resistance (IR), and diabetes [15].

The exact mechanisms by which type 1 diabetes (T1D) and poor glycemic control relate to the GH-axis and its interaction with IGF-1 and IGFBP-3 remain to be determined. Nambam and Schatz have shown that GH insensitivity in combination with low concentrations of IGF-1 is frequently observed in T1D patients [16]. However, controversies have been described in diabetes complications such as diabetic retinopathy (DR) [17]. According to Bazzaz *et al*., growth factors are associated with the development of DR, diabetic nephropathy (DN), and diabetic neuropathy (DNP). However, this article showed that growth factors, including vascular endothelial growth factor (VEGF), IGF-1, and tumor necrosis growth factor, may have a protective role in the progression and development of diabetes complications [18].

The pathogenesis of DR is a complex process involving ischemia and hyperglycemia; growth factors may result in neovascularization and loss of vision. There is controversy about the serum IGF-1 level that correlates with the progression of retinal neovascularization in clinical diabetes and increased or decreased concentrations of IGF-1 in the vitreous or serum levels of patients with DR [19]. Neam u *et al*. showed that decreased concentrations of IGF-1 were positively correlated with diabetes and diabetes-related complications [20].

Evidence has suggested that patients with T1D may have aberrations of the GH/IGF/IGFBP axis, including GH hypersecretion, decreased concentrations of circulating IGF-1 and IGFBP-3, and elevated levels of IGFBP-1 [12]. These abnormalities may exacerbate hyperglycemia in patients with T1D and play a role in the pathogenesis of diabetes-related complications [21]. Also, IGF-1 deficit has been reported to be significantly associated with the risk of developing impaired glucose tolerance, IR, and type 2 diabetes mellitus (T2D) [22]. The study also suggested that IGF-1 deficit may be a factor in the pathogenesis of schizophrenia [22]. Knott in 1998 presented a clear association between high levels of IGF-1 and the progression of DR [23]. Therefore, the aim of this review was to discuss a large body of evidence on the therapeutic potential of human IGF-1 in diabetes complications and to understand the mechanism and metabolic links of IGF in diabetes complications.

## 2 The physiology of the IGF system

The IGF system is encompassed of IGFs, IGF-1 and IGF-2 receptors, IGFBPs and IGFBP-specific proteases [9]. Roith *et al*. stated that IGF decreases the chance of developing diabetes, cancer, and malnutrition [24, 25]. The IGF regulatory systems in different organs are tissue-specific, including liver, kidney, heart, and other tissues, but all share similar components [26-28].

IGF-1 and IGFBP-1 are regulated by pituitary GH and IGFBP-3. IGF-1 and IGFBP-3 form complexes that bind to the acid-labile subunit (ALS) and prevent the premature degradation of IGF-1 by circulating IGF-1 proteases. Its release into the extravascular space extends the half-life of IGF-1 and initiates the transport into specific target tissues [16]. Aguirre *et al*. reported that IGF-2 has similar physiological properties to IGF-1, and its actions have remained poorly characterized, but the appropriate roles in fetal growth and development and cerebral protection have been documented well [29].

### 2.1 Physiology of insulin and IGFs

Insulin is secreted by β-cells in the pancreas. It has both endocrine and exocrine function. Insulin consists of two peptide chains, “A” and “B”. The other “C”-peptide is formed and cleaved off when pro-insulin transforms to active insulin. Insulin increases glucose uptake in muscle and fat by stimulating the translocation of glucose transporter 4 from the cytoplasm to the cell surface, but inhibits glucose production in the liver. It also stimulates lipogenesis, glycogen, protein synthesis, and cell growth and differentiation. The functional defect and deficiency of insulin both cause diabetes to develop accompanied by elevated fasting and postprandial glucose levels and elevated free fatty acid levels [10].

The GH-IGF axis requires the hypothalamic pituitary axis for production of GH, its receptor for IGF production, IGFBP for transport of IGF, and IGF receptor for IGF action [30]. Laron *et al*. suggested that IGF-1 acts as an endocrine hormone that is secreted by hepatocytes and transported to other tissues [31]. They also found that it is secreted by other tissues as well including cartilaginous cells, acts locally as a paracrine hormone, and may act in an autocrine manner in oncogene [31]. IGF and insulin are proteins with high amino acid sequence similarity. They have structural similarity, but control different aspects of growth, development, and metabolism. IGF-1 and insulin fully activate IGF-1R and insulin receptor, but they can also interact with each other and activate the other receptor with low affinity [17, 26, 32, 33].

### 2.2 IGF-1 and IGF-2

IGF-1 is a peptide hormone consisting of 70 amino acids with an “A “and a “B” chain which are connected by disulphide bonds and with the “C”-peptide region [31, 34]. The IGF-1 gene is under GH control in certain tissues such as liver, kidney, and heart, but is also responsive to factors like developmental signals, nutritional status, diabetes, aging, and neural activity. The IGF-2 gene is also responsive to all the above conditions with the exception of GH. IGF-2 binds to the IGF-1 receptor; it is believed that most of the actions of IGFs take place through its receptors [26, 30, 32]. Bachner-Melman has demonstrated the functions of growth factors and stated that they play a role in the mediation of GH action, stimulation of the growth of cultured cells, and stimulation of insulin action; they are involvemeed in both development and growth [35]. IGF-1 is necessary for normal insulin sensitivity; impairment in IGF-1 synthesis results in IR. IGFs in biological fluids are accompanied by IGFBPs which are the major regulators of IGFs’ biological activity and metabolic signaling pathways [26].

### 2.3 Interactions of insulin receptor and IGFs

Both insulin and IGF-1 have their own receptors, namely insulin receptor and IGF-1R, which originate from the same family of tyrosine kinase receptors [10]. Lewitt *et al*. stated that IGFs interact with insulin receptor A and B isoforms, IGF-1R, and hybrid receptors, including insulin receptor A-IGF-1R and insulin receptor B-IGF-1R. This interaction is used to mediate signals in various tissues in order to harmonize protein, carbohydrate, and fat metabolism. Interestingly, liver cells and mature adipose tissue cells have ample insulin receptor B, and insulin has a 2-fold higher affinity for insulin receptor B than IGF-1 [15].

The IGF-1 receptor has been found in various body systems, including brain, testes, liver, and bones. This suggests an important paracrine and endocrine role of IGF. Insulin binds to the IGF-1 receptor with lower potency compared to IGFs, and IGF binds to the insulin receptor to activate the reduction in blood glucose levels in the body [36]. IGF-2R binds to IGF-2, almost exclusively, and plays a minor role in the growth-promoting effect of IGF [30, 31].

A previous study reported that nutrition and GH stimulate the synthesis of IGF-1 in liver and other tissues. The study revealed that there are gender differences in the hepatic sensitivity to GH, and that women require more GH to synthesize IGF-1 in liver and other tissues [37]. IGFs that reach the pituitary hinder GH synthesis in a feedback loop. GH has an imperative metabolic role independent of IGF-1 effects, stimulation of lipolysis, and inhibitory effects on insulin signaling in fat and muscle cells [38]. Thus, IGF feedback inhibition of GH by dropping the direct metabolic effects may improve insulin sensitivity. Dynkevich *et al*. concluded that IGFs directly control protein, carbohydrate, and fat metabolism, and IGF-1 also augments insulin sensitivity independent of its consequence on GH [39].

### 2.4 Insulin-like growth factor-binding proteins (IGFBPs)

The IGFBP family is a critical component of the IGF system; it controls the biological actions of the IGFs and may also be capable of IGF-independent actions [33]. Back *et al*. have reported IGFBPs to be regulators of growth factor bioavailability by forming IGFBP-IGF complexes [10]. According to Adamek *et al*., IGFBPs are also used to supply IGFs in specific tissue sections, restrict the activity of IGFs by depressing the availability of their receptors, and shield them from proteolytic degradation [26]. Moreover, soluble IGFBPs are specific proteins that are capable of interacting with IGFs in extracellular and interstitial fluids of living organisms [40]. In the plasma, 99% of IGFs interact with the family of compulsory proteins, which controls the accessibility of free IGF-1 (fIGF-1) to the tissues. In humans, nearly 80% of circulating IGF-1 is transported by IGFBP-3, a ternary complex comprising one molecule of IGF-1, IGFBP-3, and ALS each [31, 32].

Unbound IGFs and IGFs in binary interactions have short lifespans, they are estimated to last minutes to hours in the circulation. Total IGF estimation in single blood specimens consequently undervalues this dynamic IGF turnover and fails to show the appropriate tissue IGF production, which contributes to the activity of IGF at the cellular level [33, 41].

Another study has reported that IGFBP-1 concentrations are repressed in response to increased insulin levels in obesity, and that low IGFBP-1 concentrations forecast the development of T2D. Visceral adiposity and hepatic steatosis, along with long-lasting inflammation, contribute to the IGF system phenotype in persons with metabolic abnormalities and T2D. The IGF system participates in vascular pathophysiology and other complications and may therefore be a potential therapeutic target [15]. Importantly, the incidental effects of IGF-1 that impact metabolism include blockade of GH and insulin secretion.

The activities of IGF-1 are controlled by IGFBPs. In obesity and metabolic syndrome (MetS), there is foremost dysregulation of IGFBP secretion resulting in changes in the levels of free IGF-1. In T1D, IGF-1 synthesis is significantly reduced, while in T2D various deviations arise in IGF-1 actions such as sensitization to its mitogenic actions in some target tissues, including liver, pancreas, and peripheral tissues [42].

## 3 IGFs, insulin resistance (IR), and diabetic complications

### 3.1 IGFs and IR

IR can be defined as a state in which target tissues show a reduction in responsiveness to insulin. Evidence showed that in T1D a reduction in insulin levels in the portal vein results in dysregulation of the GH/IGF/ IGFBP axis [43]. T1D has been associated with hepatic GH resistance and increased production of IGFBP-1 and -2. Decreased levels of IGFBP-3 result in reduced levels of circulating IGF-1 [10, 43].

Pancreatic β-cells secrete insulin in response to an augmented blood glucose level to compensate for the IR state; β-cells increase basal and postprandial secretions of insulin. However, the cells can no longer compensate for IR and fail to respond appropriately to the impairment in glucose disposal [44], which leads to distorted glucose homeostasis and development of hyperglycemia. In turn, the state of hyperglycemia impairs peripheral IR and insulin action [44, 45].

IR is a pathological condition also resulting in decreased efficiency of insulin signaling for blood glucose regulation [44]. It is a key component of MetS. It also increases the risk of various diseases including T2D, cerebrovascular damage, coronary artery disease, and neurodegenerative disorders [46].

Genetic disorders of IR are characterized either by mutations affecting the insulin receptor or defects in post-receptor sites. For example, T2D is associated with the downregulation of peripheral insulin-binding sites and upregulation of tissue-specific IGF binding [21]. Furthermore, evidence suggests that IR, along with the associated hyperglycemia, occurs in classic insulin target organs, and that these conditions are the pathological hallmark of metabolic disorders such as obesity and T2D [46].

A study has investigated the effect of IGF-1 on insulin sensitivity and its relation to T2D. The National Health and Nutrition Examination Survey III reported a higher risk of IR, MetS, and T2D in patients with low serum IGF-1 level [21].

A mice model showed that deletion of hepatic IGF-1 production may result in 80% reduction in IGF-1 concentration and subsequently increased insulin concentration in the blood as well as disorders in blood glucose concentration and glucose clearance [47, 48]. Moreover, supporting evidence by Friedrich *et al*. showed a negative relationship between IGF-1 levels and IR measured by the homeostasis model assessment of IR [49].

Evidence suggests that the IGF axis may play a role in glucose homeostasis. Rajpathak *et al*. showed that exogenous administration of IGF-1 decreases serum glucose concentrations and improves insulin sensitivity in individuals with and without T2D [34]. Also, insulin and IGF-1 are capable of increasing glucose uptake and glycogen synthesis and decreasing protein catabolism [42]. IGF-1 has little effect on the adipocyte and mature liver due to a lack of IGF-1 receptors at these sites, but recombinant human IGF-1 has been shown to overwhelm hepatic glucose production via unknown mechanisms, and recombinant human IGF-1 therapy has proved efficacious in patients with severe IR [50].

Deviations in GH and IGF-1 function alter insulin’s ability to maintain normal carbohydrate homeostasis [42]. In a mice model, elimination of IGF-1 synthesis in the liver and crossbreeding with mice that overexpress a mutant form of GH that prevents GH activation of its receptor showed that GH is a major determinant of IR in IGF-1-deficient mice [51].

Administration of IGF-1 to normal humans resulted in lower concentrations of glucose; the effect was nearly 1/10th as potent as that induced by insulin. Supporting evidence by Clemmons *et al*. showed that patients with extreme IR had improved insulin sensitivity and carbohydrate homeostasis after IGF-1 administration [51].

**Figure 2.**
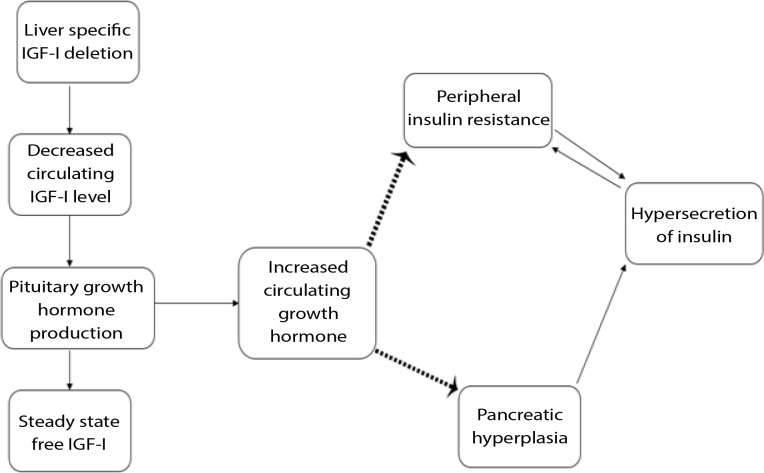
Liver derived circulating IGF-I in muscle insulin sensitivity. Liver-specific IGF-I gene deletion is associated with a noticeable decrease in circulating total IGF-I levels and elevated GH levels. Subsequently, insulin insensitivity at the level of the muscle as well as islet cell hyperplasia are associated with hyperinsulinemia (Adapted from Friedrich, *et al*., [49])

In summary, hepatic IGF-1 production plays a role in the reduction of IGF-1 concentrations, which directly increases concentrations of insulin in the blood and results in elevated blood glucose concentrations and IR [47-49] (Figure 2).

### 3.2 The metabolic link between IGFs and diabetes complications

Insulin regulates cellular energy supply and macronutrient balance and direct anabolic processes of the fed state. It is crucial for the intracellular transport of glucose into insulin-dependent tissues, including skeletal muscle, adipose tissue, and liver [52]. Similarly to insulin, IGF-1 also promotes protein synthesis in skeletal muscles and other tissues. Insulin also appears to have another impact on the metabolism in vascular smooth muscles as there are only receptors for IGF-1 in these tissues [53].

In humans, a study showed that insulin upregulates hepatic GH receptor expression and increases net cell surface receptor availability in the portal circulation. Although GH and insulin have metabolically opposed hormones, insulin has been described to play a role in facilitating the action of GH. In children with T1D, low concentrations of GH-binding protein secondary to low levels of portal insulin have been reported, which indirectly decrease levels of IGF-1 [16]. The decreased level of IGFBP-1 in T1D may be caused by absolute insulin deficiency. A study in T2D patients suggested that decreased IGFBP-1 concentrations were due to hyperinsulinemia [11, 14].

Insulin levels decrease during fasting to enable mobilization of fatty acids, glycerol from adipose tissue, and amino acids from muscle, but they increase in the fed state [52]. The extent of insulin sensitivity may be predisposed by the composition of the diet, and chronic surplus energy consumption endorses hyperinsulinemia. IR triggers complete stimulation of insulin secretion, triglyceride synthesis, and fat buildup, while insulin receptors are downregulated [53]. Prospective data showed that low levels of IGF-2 may induce the risk of weight gain in T2D patients. This strong inverse association seems to be independent of other risk factors for weight gain and obesity [54].

The effects of fasting on vascular smooth muscle metabolism appear to be similar to the effects of diabetes on vascular metabolism during the early stages of diabetes. The relation of high-fat diets and IR seems to be due to saturated fat and trans-fatty acids because these fatty acids play a role in the development of IR through effects on the composition of membrane lipids [55].

GH acts on IGF-1 secretion, which has metabolic actions of its own and depends on weight status. A study showed that IGF-1 is dependent on body mass index (BMI), with a maximal level at BMI of 30-35 kg/m^2^. This relation is reflected in severe GH deficiency, indicating that GH-independent IGF-1 secretion represents an imperative metabolic regulator [56].

Infusion of recombinant human IGF-1 in IR patients showed that IGF-1 plays a role in the regulation of cell mass, insulin secretion, and regulation of insulin sensitivity. The energy-sensing character of a cohesive IGF-1/insulin system controls lipolysis, proteolysis, and IR [56]. Moreover, administration of IGF-1 to patients with IR showed an improvement in glycemic status. Clemmons *et al*. reported that IGF-1 is associated with lowering blood glucose concentrations and increasing insulin sensitivity in diabetes patients. However, diabetes patients are also sensitive to stimulation of adverse effects in response to IGF-1. IGF-1 coordinately links GH and insulin action and has direct effects on intermediary metabolism [42].

Other studies reported that IGF-1 impacts lipid and glucose metabolism [57], and that its exogenous administration augments insulin sensitivity in healthy adults and T2D patients. Sesti *et al*. showed that in about 500 patients IGF-1 concentrations were autonomously associated with insulin sensitivity, accounting for 10.8% of its variation [58]. IGF-1 plasma concentrations were associated with a 90.5% decrease in the risk of MetS [58].

In summary, GH, IGF, and insulin have important roles in normal physiology of the body. GH and insulin are metabolically opposed hormones. Insulin has been described to have a permissive role in facilitating the action of GH, and relatively low levels of IGF may increase the risk of weight gain in diabetes patients. Administration of IGF-1 to patients with IR may improve their glycemic status and positively impact lipid and glucose metabolism.

### 3.3 IGFs and their association with diabetes complications

IGF-1 has anti-inflammatory and pro-survival effects on the vasculature, resulting in decreased vascular oxidant stress, apoptosis, and inflammatory signaling. Reduced IGF-1 activity has been shown to endorse cerebro-microvascular dysfunction, accelerate endothelial apoptosis, and diminish the regenerative ability of the endothelium [59].

Low IGF-1 levels have been shown to forecast glucose intolerance and T2D. fIGF-1 concentrations are linked with the occurrence of diabetes in women with insulin concentrations beyond the median, but below median IGFBP-1 concentrations. However, poorly managed T2D patients exhibit an increase in IGFBP-3 glycation, which raises the affinity for IGF-1, and additional sialylation that has the opposite effect, namely to reduce IGF-1 affinity [15].

Evidence is emerging that dysregulation of the IGF system is involved in the development of diverse complications of diabetes. IGFs, specifically IGF-2, have sturdy anti-apoptotic effects that are present in the proliferation of endothelial cells and numerous cancer cell lines. It has been argued that regional concentrations of IGFs may be more substantial than systemic concentrations in the pathogenesis of diabetes complications [11, 17].

Clinical surveys have verified that reduced circulating levels of IGFBP-1 are linked to T2D, while high serum IGFBP-1 levels are related to T1D. Serum IGFBP-1 concentrations are found to be augmented in T1D patients with microalbuminuria [14].

IGFBP-3 is regulated by GH, whereas IGFBP-1 is regulated by insulin, which downregulates the production of IGFBP-1. Portal hypoinsulinization of T1D leads to higher amounts of IGFBP-1, which decreases accessible bioactive fIGF-1. These effects are the concomitant chronic inflammation accompanying T1D. Higher levels of pro-inflammatory cytokines, such as tumor necrosis alpha and interluikin-1β, induce IGF-1 resistance. A study has shown that higher levels of interleukin 8 in T1D are related to lower IGF-1 levels in adolescents [16]. Hedman *et al*. in 2004 also studied the GH/IGF-1 axis in adults with long-standing T1D [16]. They speculated that tIGF-1 and fIGF-1 would be higher while IGFBP-1 would be lower. They showed that low IGF-1 concentrations may cause poor growth in children and play a role in IR and poor metabolic and cardiovascular outcomes [16].

In general, systemic administration of recombinant human IGF-1 may reduce the levels of insulin and glucose in diabetes patients with reduced response to insulin, which indicates that IGF-1 is capable of increasing insulin sensitivity [50] and has broad effects on IR and hyperglycemia.

### 3.4 IGF and retinopathy

Diabetic retinopathy (DR) is a complication of diabetes that affects the eyes. It results primarily from harm to the blood vessels triggered by hyperglycemia. DR results in the gradual loss of sight leading potentially to blindness. Nearly all people with T1D and more than half with T2D develop complications involving the retina [17].

Hyperglycemia leads to numerous biochemical aberrations which eventually cause retinal ischemia and vulnerability to dysregulated angiogenesis. Growth factors are upregulated along with their individual receptors, and disparity between pro-angiogenic and anti-angiogenic factors leads to new blood vessel formation in the retina. Vessels are predisposed to outflow and hemorrhage because of the friable nature of the newly formed blood vessels and insufficient cell-to-cell junctions, which may finally result in retinal detachment [17, 32].

The alterations in growth factors are believed to be significant in both initial and late phases of DR. Loss of pericytes and injury of endothelial cells are two factors that may exacerbate growth factor-mediated proliferative response. These effects contribute to increased endothelial cell matrix deposition and basement membrane thickening, leading to proliferation and migration of endothelial cells and consequent neovascularization [17]. Moreover, failure of the blood-retinal barrier (BRB) is another feature of DR similar to venous occlusive diseases and cystoid macular edema. Intravitreal administration of IGFBP-3 conserves junctional stability in the presence of VEGF or laser injury by reducing BRB permeability in part by modulating sphingomyelinase levels [60].

A previous study has verified that circulating concentrations of t/fIGF-1 are low in patients with T1D compared with age- and sex-matched controls. Age-adjusted fIGF-1 levels were much higher among diabetes patients with DR than in those without DR [21], but another study found no real connection between IGF-1 levels and the progression of retinal disease [23].

The risk factors that are believed to be related to DR include diabetes as well as high levels of glycated hemoglobin, blood pressure, low-density lipoprotein cholesterol (LDL-C), and urinary microalbumin. The clear pathogenesis of DR was not questioned by the authors of a study on DR and IGF, but according to the report, GH and IGF-1 may also be involved in DR [61].

The role of IGF-1 in DR and other diabetes complications has been extensively studied. However, there are inconsistent results as to the relationship between IGF-1 and the clinical stage of DR. It is considered that autocrine and paracrine tissue production of IGF-1 may contribute to the variation in correlation studies [17].

The development of adjuvant treatments to enhance diet, exercise, and the use of oral hypoglycemic agents and insulin is definitely desirable to improve the quality of patient life. The neurobiology of IGFs has been investigated in animals, and it was found that a loss of IGF activity may produce neurological syndromes that mimic the disorders of DR [32]. Moreover, insulin is the most effective hormone for the treatment of hyperglycemia by mediating direct and indirect actions to lower hyperglycemia. The application of IGF-1 could be considered when an extreme IR or insulin insensitivity exists, while recombinant human IGF-1 might be an alternative strategy to normalize the blood glucose level and prevent acute complications of diabetes [62].

In summary, the GH-IGF-1 axis in the development and progression of DR has been studied for decades since surgical removal of pituitary (hypophysectomy) was first introduced as a treatment for DR. Rapid improvements in glycemic control can promote clinical regression of proteinuria and symptomatic neuropathy. These changes may also be caused by a momentous deterioration of retinal disease, because improvement in glycemic control is associated with an increase in serum IGF-1 levels. IGF-1 has also been shown to act as an angiogenic agent in animal cornea and retina [21].

### 3.5 IGF and cardiovascular disease

Cellular senescence as well as impaired vascular endothelial proliferation, adhesion, and incorporation play critical roles in the occurrence of macrovascular disease [63]. Higher IGF-1 bioavailability may protect the onset of ischemic heart disease and glucose intolerance in T2D patients, thus improving metabolic control and preventing vascular complications. Other likely beneficial actions of IGF-1 in cardiovascular physiology include augmented nitric oxide synthesis and potassium channel opening [64]. This may explain the weakened small vessel function associated with low IGF-1 levels in patients with cardiovascular disease (CVD) through LDL cholesterol-activated cytotoxicity and vascular smooth muscle cell apoptosis. IGF-1 possibly also protects against plaque instability and rupture [49]. In patients with acute myocardial infarction, noticeably reduced IGF-1 levels are linked with a poor outcome. The authors reported that intramyocardial or vascular gene delivery of growth factors can progress symptoms in patients with coronary or peripheral vascular disease [65].

Importantly, low IGF-1 concentrations are associated with late mortality in patients with myocardial infarction, cardiac failure, and diabetes. Interventional studies suggest that IGF-1 has antiatherogenic actions because of its multifactorial effect on CVD and related risk factors. The antiplatelet and antithrombotic effects of IGF-1 are crucial effects in avoiding both vascular damage and unstable coronary plaques [66].

Short-term anti-inflammatory properties of IGF-1 seem to decrease infarct size and promote left ventricular remodeling after myocardial infarction. Furthermore, IGF-1 also has an immune modulatory activity that may inhibit autoreactivity. This makes IGF-1 an important protein explaining the antithrombotic and anti-remodeling activities [66].

IGF-1 increases the contractility of cardiomyocytes mainly by increasing intracellular calcium level and calcium sensitization of myofilaments as well as by conserving capillary density. GH and IGF-1 are likely to increase protein synthesis in the cardiomyocytes. Reuptake of calcium is also promoted by IGF-1 through the regulation of the sarcoendoplasmic reticulum Ca^2+^-ATPase (SERCA2) which is involved in diastolic function [67].

The IGF-1 system, especially low IGF-1, low IGFBP-1, and high IGFBP-3, is related to increased CVD risk. The production of IGFBP-1 in the liver is downregulated by insulin. There is a correlation between low levels of IGFBP-1 and hyperinsulinemia, which may also be linked with increased CVD risk. However, IGFBP-1 levels rise during the development of T2D, regardless of persisting hyperinsulinemia, indicating increased hepatic IR during CVD progression [68].

In a randomized trial of dilated cardiomyopathy, GH had neutral effects on heart function and symptoms. However, the concentration of IGF-1 induced by GH rather than GH itself affects the improvement in heart function. IGF-1 has proliferating, inotropic, vasodilator, and antiapoptotic effects in the cardiovascular system. Acute injection of IGF-1 into apparently healthy individuals and cardiac failure patients resulted in inotropic effects [69]. Furthermore, a study assessed the relationship between congenital heart disease and underweight in infants. The authors reported that underweight infants with congenital heart disease had a significantly reduced energy intake and substantially low serum IGF-1 and IGFBP-3 levels. The finding suggested that low levels of IGF-l and IGFBP-3 were observed in nutritional deficiency. The authors recommend that consecutive measurements of serum IGF-l and IGFBP-3 may be supportive in monitoring the effect of nutritional supplement in congenital heart disease [70].

Therefore, IGF-1 levels have substantial associations with CVD; low concentrations are associated with myocardial infarction and cardiac failure. Measurement of IGF levels in patients with CVD may improve the prognosis and predict the outcome of the disease. Administration of IGF-1 may lead to an increase in the production of nitric oxide, which may result in a reduction of the systemic vascular resistance.

### 3.6 IGF and neuropathy

Diabetic neuropathy (DNP) is a disorder subclinically or clinically evident in both peripheral and autonomic nervous systems [71]. Neuropathies are the foremost shared complication of diabetes affecting up to 50% of patients with T1D. Distal polyneuropathy becomes symptomatic after several years of diagnosis in T1D; in T2D patients, neuropathy is detected at the time of diagnosis [72].

It is generally thought that diabetic neural instabilities may be secondary to hyperglycemia, but this remains a debatable issue. IGFs are neurotrophic factors capable of supporting neurite outgrowth and endurance in peripheral and central neurons [73].

Insulin, IGF-1, and IGF-2 are proposed to provide neurotrophic support for neurons. In diabetes, IGF activity is reduced [74]. Neuropathy can be prevented by administration of IGF. Central and peripheral neurological disturbances both share a common etiology involving IGF, and they are treatable irrespective of hyperglycemia [32].

The mechanisms by which diabetes causes central nervous system complications are not clear. In the central nervous system, apoptotic neuronal cell death has been designated in ischemic brain injury and neurodegenerative diseases [75], including Alzheimer’s disease and others. Furthermore, a study on duration-dependent neuronal apoptosis in the hippocampus of T1D rats found that neuronal loss and cognitive impairments are associated with duration of diabetes [28].

In the peripheral nervous system of T1D patients, IGF-1, insulin, and C-peptide contribute to the development of axonal degenerative changes and participate in weakened regenerative capacities [75]. In T2D, IGF aberrations are less noticeable, which may in part account for the milder neurological complications in T2D patients. Therefore, renewal of several members of the IGF system may offer a realistic chance for prevention and treatment of DNP [28].

IGFs have been shown to stimulate motor neuron proliferation and differentiation. They increase motor neuron myelination, inhibit demyelination, reduce neuron apoptosis during normal development, and enhance axonal regeneration after injury. They also protect neurons from toxicity induced by chemicals, cytokines, and cancer chemotherapy. T1D is characterized by both a decrease in tIGF-1 levels and an increase in sequestration of IGFs by higher levels of IGFBP-1 resulting in further decrease in bioavailable free IGFs [21]. Also, in a mice model of T2D with peripheral neuropathy, lower serum levels of IGF-1 and red cell IGF-1 receptor were detected than in non-neuropathic mice with diabetes and non-diabetes controls [21].

Therefore, in DNP, numerous metabolic and vascular changes are interconnected to cause damage to nerve cells in a similar way to that observed in DR with the primary underlying feature being hyperglycemia. The changes include increased oxidative stress, formation of advanced glycation end-products, increased activity of the polyol pathway, activation of pro-inflammatory mechanisms, and ischemia. These pathways have direct and indirect adverse effects on neurons and blood vessels that provide blood supply to the nerves, particularly in diabetes patients [76].

### 3.7 IGF and nephropathy

Diabetic neuropathy (DN) is a major cause of morbidity and mortality in patients with T1D and T2D. About one third of this population suffers from this long-term complication of diabetes, and diabetes has become the leading cause of end-stage renal disease (ESRD) worldwide [77].

DN is a clinical syndrome detected by albuminuria and continuous decline in the glomerular filtration rate [78]. Usually, patients with T1D develop ESRD earlier than those with T2D [79]. The early physiologic abnormality in DN is that glomerular hyperfiltration is related to intra-glomerular hypertension, and this is complemented by the onset of microalbuminuria [79].

The kidney is a source of significant synthesis and target for IGF-1 action [80]. *In vitro* IGF-1 has been shown to be mitogenic for renal cells, promote nephron hypertrophy, and stimulate tubular phosphate transport. Supporting evidence by Thrailkill *et al*. has shown that systemic administration of IGF-1 causes increased renal blood flow and increased glomerular filtration rate [21]. Therefore, the IGF system is an important constituent for the normal functioning of the kidney and enhancing repair of an injured kidney; dysregulation of the system has been reported in a variety of kidney diseases.

## 4 Clinical/therapeutic potential of IGFs for diabetes mellitus and its complications

Growth factors are generally implicated in the development of DR and DN. In DNP, VEGF, IGF-1, and necrosis growth factor have protective roles. These different effects of growth factors may also be partly related to the discrete pathophysiological nature of different endpoints [18].

Injection of IGF-1 in patients who develop IR leads to improved glycemic control. IGF-1 has been shown to be linked with a glucose-lowering effect and increasing insulin sensitivity. However, diabetes patients are sensitive to the stimulation of side effects in response to IGF-1, which limits the practicality of IGF-1 as a hypoglycemic agent [42].

IGF-1 serum levels have not usually been found to be increased in diabetes patients. Instead, there is IGF-1 reduction at the systemic level which is most pronounced in patients with poor glycemic control. At the tissue level, IGF-1 availability is decreased because of reduced serum f/tIGF-1 and increased IGFBP-1 (which is an inhibitor of IGF-1). During diabetes monitoring and follow-up to improve glycemic control, the serum level of IGF-1 usually increases. This phenomenon explains the pathophysiology of the “bush fire” which is a brief exacerbation of proliferative DR following better glycemic control. Hence, the relationship of retinal ischemia with local IGF-1 and IGF-2 production, IGFBPs, and angiogenesis has been recognized [18].

The availability of recombinant human IGF-1 has the potential to use the peptide in the treatment of a variety of disease states. In the literature, T1D has been emphasized because of the relative portal insulin deficiency. This disorder is thought to be accountable for the decreased circulating levels of IGF-1 [81].

Improved glycemic control has been described in patients with T1D and T2D after IGF-1 treatment. IGF-1 administration reduces GH hypersecretion of adolescents and adults with T1D. The reductions in GH secretion were associated with decreased insulin requirements without alteration in glycemic control, which indicates an increase in insulin sensitivity [81]. The authors have also explored the effects of IGF-1 infusion on glucose and protein metabolism in healthy individuals. They found that large doses of intravenous IGF-1 have effects comparable to insulin on glucose metabolism, but the effects on protein metabolism are different and are characterized by decreasing circulating amino acid levels [81].

Oral monotherapies did not achieve the target of intensive therapy in T2D patients. The combination of insulin, oral agents, and diet can partially reduce diabetes complications. Intensive therapy reduced glycated hemoglobin levels by approximately 1%, which is below the results achieved by conventional therapy resulting in a 35% reduction in the risk of developing diabetes complications [32].

The effects of short-term administration of recombinant human IGF-1 on glycemic control and control of the GH-IGF-IGFBP axis in patients with T1D have been reported [21]. The study showed that a single subcutaneous injection of recombinant human IGF-1 (40 mg/kg) resulted in elevated levels of IGF-1, reduced overnight secretion of GH, and declined insulin necessities in 9 pubertal adolescents with T1D [21]. Moreover, both insulin and IGF-1 are able to provoke glucose uptake, glycogen synthesis, and inhibition of protein breakdown. IGF-1 has little effect on adipocytes and hepatocytes because of the lack of IGF-1 receptors, but recombinant human IGF-1 has shown to inhibit hepatic glucose production via unknown mechanisms [50]. Therefore, recombinant human IGF-1 therapy in patients with IR may be effective.

## 5 Conclusions and recommendations

The IGF system is a very important endogenous mechanism recruited daily for beneficial action in cardiovascular and metabolic health disorders, IR, and diabetes complications. The role of IGF-1 in the development of IR and diabetes complications has already been described. An inverse relationship between the circulating levels of IGF-1 (IGF-ItoIGFBP-3 ratio) and MetS, diabetes, and CVD has been reported. It may thus be considered that low circulating levels of IGF-1 are associated with the development of MetS and raise the risk of CVD and diabetes complications.

Cellular senescence reduces vascular endothelial proliferation. Adhesion plays an essential role in the progression of macrovascular disease. High IGF-1 availability may defend against the onset of CVD and glucose intolerance in diabetes patients. Moreover, IGF-1 increases nitric oxide production and potassium ion channel opening in cardiovascular physiology, both of which improve the weakened small vessel function linked with low IGF-1 concentrations in patients with cardiovascular syndrome.

Therefore, the IGF system is involved in vascular pathophysiology during progression to and protection from vascular complications; it has also been implicated in metabolic abnormalities. Therefore, the IGF system may have a therapeutic potential in reducing the risk of the development and progression of vascular complications in diabetes patients. Further studies should focus on the role of IGF in diabetes complications and emphasis the mechanism of the system to tackle disease progression and reduce diabetes-related complications in large cohorts of patients.

## Abbreviations

ALS acid-labile subunit

BRB blood-retinal barrier

CVD cardiovascular disease

DN diabetic nephropathy

DNP diabetic neuropathy

DR diabetic retinopathy

ESRD end-stage renal disease

fIGF free insulin-like growth factor

GH growth hormone

GHR growth hormone receptor

IGF insulin-like growth factor

IGF-1R/2R insulin-like growth factor 1 receptor / 2 receptor

IGFBP insulin-like growth factor binding protein

IR insulin resistance

LDL low-density lipoprotein

MetS metabolic syndrome

T1D type 1 diabetes mellitus

T2D type 2 diabetes mellitus

VEGF vascular endothelial growth factor
